# “Primary Hyperparathyroidism (PHPT) in Children: Two Case Reports and Review of the Literature”

**DOI:** 10.1155/2021/5539349

**Published:** 2021-04-13

**Authors:** Gerdi Tuli, Jessica Munarin, Daniele Tessaris, Raffaele Buganza, Patrizia Matarazzo, Luisa De Sanctis

**Affiliations:** ^1^Department of Pediatric Endocrinology, Regina Margherita Children's Hospital of Turin, Turin, Italy; ^2^Department of Sciences of Public Health and Pediatrics, University of Turin, Turin, Italy

## Abstract

Primary hyperparathyroidism (PHPT) is a rare disorder in children and adolescents. Typical biochemical features are hypercalcemia and hypophosphatemia, but the clinical features can be heterogeneous, and in some cases, symptoms are vague and nonspecific, leading to misdiagnosis or late diagnosis. Herein, we report two cases of PHPT in pediatric age with different presenting symptoms, pain in the foot, and progressive alteration of the gait in the first case and recurrent abdominal pain with emotional lability in the second. Biochemical and radiological assessment confirmed PHPT. Both cases were treated surgically as definitive treatment, but in the second case, previous medical treatment with cinacalcet, a calcimimetic agent, was required to reduce serum PTH and calcium levels. After surgery, despite conventional treatment with calcium and calcitriol, case 1 developed a hungry bone syndrome. The analysis of the MEN-1 (Multiple Endocrine Neoplasia) gene was negative in both cases. A diagnosis of PHPT should be considered when children or adolescents present bone pain with radiological imaging of osteolytic lesion and biochemical feature of hypercalcemia associated with hypophosphatemia. In PHPT, the gold standard treatment is represented by surgery followed by strict postoperative endocrine monitoring to maintain adequate homeostasis of calcium and bone metabolism.

## 1. Introduction

Primary hyperparathyroidism (PHPT), a rare disorder in pediatric age, with an estimated incidence of 2–5 cases in 100,000 live births, is characterized by excessive secretion of parathyroid hormone (PTH), responsible for hypercalcemia and hypophosphatemia [1–3]. Most cases of PHPT are not hereditary, and the familial PHPT accounts for 10–20% of cases [4–7].

The main clinical features involve the skeleton and the kidney. Bone pain, skeleton deformities, and the increased fracture risk at any skeleton sites are the most important symptoms. Rarely, the first manifestation of PHPT can be rickets or osteomalacia, associated with short stature [1,7]. Nephrocalcinosis and nephrolithiasis are the main complications of PHPT, and hypercalciuria is the most common risk factor for renal stone development.

Other clinical features related to PHPT include neurological symptoms (attention-deficit disorders, irritable behaviours, depression, weakness, seizures, proximal myopathy, and lethargy), gastrointestinal symptoms (vomiting, nausea, diarrhea, and acute pancreatitis), and cardiovascular complications, including prolonged QT interval (7–10).

Radiological assessment includes parathyroid and thyroid ultrasound and 99mTC-sestamibi scintigraphy, total body bone scintiscan, jaw X-ray, and X-ray evaluation of the skeletal site with bone pain.

The gold standard treatment in children is surgery [8, 9]. In the presence of severe hypercalcemia, parenteral isotonic solutions or calciomimetic agents such as cinacalcet, which lower serum calcium and PTH, but do not improve bone mass density (BMD), are required [10]. An alternative presurgical therapeutic option includes the use of bisphosphonates that improve BMD and lower biochemical markers of bone turnover in patients with PHPT, but have less effect on lowering calcium and PTH [7].

Similarly to adults, solitary parathyroid adenoma is the most common finding in children with sporadic PHPT [2, 11], but unlike in adult patients, PHPT symptoms are frequently present at diagnosis in pediatric age. However, in the absence of a family history of PHPT, since PHPT symptoms can be vague and nonspecific, the disease may be misdiagnosed or delayed in the diagnosis [8, 9, 11], especially in pubertal age, when rapid bone growth may unmask certain skeletal manifestations of PHPT [1, 2].

We report the clinical history of two female adolescents with PHPT who differed in clinical presentation at diagnosis, both referred to the pediatric endocrinologist 12 months after the onset of symptoms. In the first case, skeletal manifestations of osteolytic lesion at the right calcaneus were present, whereas in the second case, the main symptoms were abdominal pain and irritability.

## 2. Case Reports

### 2.1. Case 1

A 16-year-old girl of Philippine origin was referred to our department as she complained of right foot pain and progressive gait abnormalities in the last 12 months. Auxological parameters showed short stature (141.1 cm, <3° Tanner percentile), within a familial short stature context, and normal weight (48 kg, 10° Tanner percentile) and BMI (24.1 kg/m^2^).

Radiographic evaluation of the lower limb demonstrated an osteolytic lesion to the right heel and a concomitant lesion of unclear interpretation to the left cuboid bone, both then confirmed at CT scan and MRI.

Biochemical investigations revealed elevated serum PTH levels (598 pg/ml; normal range 15–57), elevated serum calcium concentration (3.02 mmol/L; normal range 2.2–2.7), and low serum phosphate levels (0.56 mmol/L; normal range 1–1.8), as shown in Table 1. The urinary calcium to creatinine ratio was 0.25 mg/mg, and renal ultrasound and ECG were normal.

Total body bone scintiscan with 99Tc-oxidronate revealed increased absorption at the right heel, consistent with a brown tumor finding.

Parathyroid ultrasound did not show any significant pathological signs, but the Tc99/sestaMIBI parathyroid scan revealed an abnormal and persistent focal retention of the tracer in the right inferior thyroid pole, consistent with the hyperactivity of the right inferior parathyroid.

Therefore, the biochemical and radiological features indicated a PHPT condition; to rule out MEN-1, pituitary function was analyzed and showed normal hormonal profile. Fasting glucose, insulin, and calcitonin levels were normal; no lipoma, angiofibroma, or jaw tumors were present; both parents and siblings had normal serum PTH levels. Genetic analysis of the MEN-1 gene was negative for mutations.

The girl underwent right inferior parathyroidectomy through a minimally invasive approach, after intraoperative evaluation of the parathyroid hormone. Histological examination confirmed the presence of an adenoma (dimension 3.3 × 1.7 × 0.5 cm, weight 3 gr). To control the hypocalcemia caused by the transient hypoparathyroidism of the remaining parathyroid glands previously inhibited by the adenoma, conventional treatment with oral administration of calcitriol (0.75 mcg/day) and calcium carbonate was started; the latter was initially given at 2 g/day and then increased up to 3 g/day for numbness; finger paraesthesia and positive Chvostek and Trousseau signs were associated with low serum calcium despite high parathyroid hormone levels (Ca 1.85 mmol/L, PTH 113 ng/ml). Subsequently, the patient no longer presented signs or symptoms of hypocalcemia; laboratory analyses showed adequate calcium-phosphate metabolism and normal serum PTH levels (31 pg/ml).

Three months after surgery, biochemical evaluation again showed an increase in the PTH value (146 pg/ml), with sightly low serum calcium levels (2.02 mmol/L), configuring a hungry bone syndrome (HBS) condition, characterized by an elevated uptake of calcium by the bones after a prolonged period of hypercalcemia due to hyperparathyroidism. The oral calcium carbonate dose was, therefore, increased to 4 g/day, with normalization of serum calcium level (2.4 mmol/L) and partial response of PTH levels (67 pg/ml).

### 2.2. Case 2

A 14-year-old girl was referred to our department, having complained of recurrent abdominal pain and emotional lability for the last 12 months. Auxological parameters showed normal height (153.7 cm, 10–25° Tanner percentile), weight (47 kg, 10–25° Tanner percentile), and BMI (19.9 kg/m^2^). Biochemical investigations revealed high serum calcium concentration (3.36 mmol/L, normal range 2.2–2.7), low phosphate level (0.6 mmol/L; normal range 1–1.8), and elevated PTH levels (320 pg/ml; normal range 15–57), as shown in Table 1.

The parathyroid ultrasound evaluation showed the presence of a hypoechoic nodule in the right inferior thyroid pole (22 × 12 mm), confirmed by the Tc99/sestaMIBI scan which revealed an abnormal and persistent retention of the tracer in the same area, therefore compatible with hyperactivity of the right inferior parathyroid.

To rule out the presence of multiple endocrine neoplasia, pituitary function was investigated and showed normal hormone secretion. Fasting glucose, insulin, and calcitonin levels were normal, and no lipoma, angiofibroma, or jaw tumors were present; parents and siblings had normal serum PTH levels. Genetic analysis for MEN-1 mutations was negative.

The urinary calcium/creatinine ratio was 0.35 mg/mg, and abdominal ultrasound displayed the presence of a small hyperechoic image (5 mm) in the upper pole of the right kidney, consistent with nephrolithiasis, although asymptomatic. Spinal X-ray showed generalized bone rarefaction, evident in all vertebral bodies, and the whole-body bone scan revealed regular ^99^Tc-oxidronate uptake.

Biochemical and radiological investigations confirmed the diagnosis of PHPT. Prior to surgery, as high serum calcium levels represented a high intraoperative risk, treatment with a calcimimetic agent (cinacalcet) was started, at 60 mg/day dose for three months, with an improvement in calcium and phosphate metabolism (Ca 2.97 mmol/L, P 0.6 mmol/L), with still elevated PTH levels (457.9 pg/mL). Since cinacalcet is an off-label drug in pediatric age, permission of the local Ethics Committee and informed consent from the parents were obtained prior to its administration.

The girl, therefore, underwent right inferior parathyroidectomy through a mini-invasive approach, with intraoperative monitoring of the PTH; histological examination confirmed the presence of adenomatous tissue (dimension 2,5 × 1.4 × 0.5 cm, weight 2.5 gr). Biochemical investigations after surgery showed a rapid decrease in calcium level (2.35 mmol/L), with lowering of PTH levels (131 pg/ml); subsequently, as the girl had lip and finger tremors with mild hypocalcemia (Ca 2.01 mmol/L), conventional treatment with oral administration of calcium carbonate (1 g/day) and oral calcitriol (62.5 mcg/day) was started, with no further signs or symptoms of hypocalcemia and normalization of calcium-phosphate homeostasis and normal level of PTH (46 pg/ml) on biochemical monitoring. Calcium treatment was stopped after one month, while calcitriol after two years; currently, after 6 years from the onset of primary hyperparathyroidism, there is no recurrence of the disease and calcium-phosphate metabolism is adequate.

## 3. Discussion

PHPT is a very rare condition among children and adolescents; Table 2 indicates the data emerging from the literature describing pediatric cohorts with at least 5 subjects [1–25, 27].

The two cases of PHPT reported here showed a different clinical onset at diagnosis, with bone pain in the first case and vague symptoms such as abdominal pain and behavioural problems in the second. Both patients were referred to the pediatric endocrinologist 12 months after the onset of symptoms.

The first presented case highlights that, in case of laboratory data suggestive for PHPT, even in the presence of US of the neck negative for pathological findings, a Tc^99^/sestaMIBI parathyroid scan has to be performed [9]. Once PHPT is confirmed, if bone pain is present, an X-ray of the syntomatic skeletal site and of the jaw and whole-body scintiscan should be performed, as well as an ECG evaluation.

Parathyroid adenoma represents the most frequent PHPT etiology in pediatric age, with higher aggressive behaviour than adults and higher serum and urinary calcium levels.

It is noteworthy that, in the presence of significant hypercalcemia, especially if associated with cardiac arrhythmia, the intraoperative risk is high; therefore, in such cases, the utilization of a calcimimetic agent, i.e., cinacalcet (Mimpara) or bisphosphonates, for short periods could allow to lower calcium levels, despite high levels of PTH, and to act in a safer surgical context, although few data on their use as calcium-lowering agents in pediatric age are present in the literature so far.

After successful removal of one or more hyperfunctioning parathyroid glands, patients with PHPT show a rapid transient decrease in serum calcium levels due to functional inhibition of healthy parathyroid glands. This hypocalcemia is generally mild, lasts a maximum of 2–4 days after surgery, and is independent of the size of the hyperactive glands [26, 28]. Conventional treatment with oral calcium and calcitriol is the gold standard for this condition. However, in case of long-standing PHPT, hungry bone syndrome (HBS) can occur, as shown in the first case presented. This term has been coined to represent the deep (Ca < 2.1 mmol/l) and prolonged (longer than four days after operation) hypocalcemia, following parathyroidectomy in severe hyperparathyroidism [29].

At the time of presentation, nearly 80% of children are symptomatic and have end-organ damage, mostly involving the bone and kidney [2]. The condition can manifest with various signs and symptoms related to hypercalcemia, involving the gastrointestinal, musculoskeletal, renal, and neurological systems; such nonspecific clinical presentation may, therefore, be responsible for misdiagnosis or delayed diagnosis. In the presence of such symptoms, pediatricians should be aware of monitoring bone metabolism, evaluating serum calcium and phosphate levels, as well as PTH, when bone pain or nephrocalcinosis is also present.

Once PHPT is diagnosed in pediatric age, genetic analysis for Multiple Endocrine Neoplasia (MEN) syndromes and PTH assessment, as well as X-ray of jaw and urinary calcium to creatinine ratio, in parents and siblings should be carried out to search for genetic or familial conditions [30].

In conclusion, PHPT is a rare disease in pediatric age that can be misdiagnosed; the presence of PHPT should be considered if children or adolescents present with bone pain or nephrocalcinosis, radiological imaging of osteolytic lesions, or vague gastrointestinal or neurological not otherwise explained.

## Figures and Tables

**Table 1:** Laboratory data of cases reported at diagnosis, after surgery, and at the last evaluation.

		Case 1	Case 2
Serum calcium (mmol/l)	Diagnosis	3.02	3.36
	After surgery	1.85	2.35
	Last evaluation	2.3	2.3

Phosphate (mmol/l)	Diagnosis	0.56	0.6
	After surgery	0.84	1.04
	Last evaluation	0.84	1.13

PTH (pg/ml)	Diagnosis	598	320
	After surgery	113	131
	Last evaluation	67	46

25-hydroxyvitamin D (ng/ml)	Diagnosis	6.8^*∗*^	16.2^*∗*^
	After surgery	—	61.9^*∗*^
	Last evaluation	—	43^*∗*^

CaU/CrU	Diagnosis	0.25	0.35
	After surgery	0.06	0.1
	Last evaluation	0.01	0.05
Adenoma weight (gr)		3	2.5

**Table 2:** Main papers reporting patients with PHPT in the pediatric age.

Author	Year	No. of pediatric patients	Main results
Allo et al. [12]	1982	53	Adenoma in 64.2% of studied patients; hyperplasia in 30.2%, overall in <18 y.o. (38%) vs. >18 y.o. (18.5%) patients
Lawson et al. [13]	1996	11	All sporadic PHPT. Delayed diagnosis in children; at diagnosis, 91% patients were symptomatic (renal stones 45%, abdominal pain 18%, learning difficulties 18%, musculoskeletal abnormalities 9%, and fatigue 9%). Mean serum Ca^++^: 3.39 mmol/l at diagnosis
Cronin et al. [14]	1996	8	Pediatric cohort with PHPT due to parathyroid adenoma, presenting mostly with hypercalcemic crisis (50%). Mean serum Ca^++^: 3.5 mmol/l at diagnosis
Loh et al. [15]	1998	22	Pediatric cohort with PHPT due to parathyroid adenoma, presenting mostly with fatigue (77%) or weakness (64%). Mean serum Ca^++^: 3.07 mmol/l; mean serum PTH 131 pg/ml at diagnosis
Harman et al. [16]	1999	33	Pediatric cohort with PHPT due to parathyroid adenoma, symptomatic in 94% of cases, mostly renal stones (7/33) and bone disease (9/33). Mean serum Ca^++^: 3.02 mmol/l at diagnosis; mean adenoma weight 0.96 gr
Hsu and Levine [17]	2002	16	Single adenomas in 11 patients; multiple-gland disease in 2 patients, including 1 with MEN2
Kollars et al. [2]	2005	52	Symptomatic in 79% of cases; end-organ damage (nephrocalcinosis or lithiasis, acute pancreatitis, or bone involvement) in 44%. Mean serum Ca^++^: 3.1 mmol/l; mean serum P 1.8 mmol/l; mean serum PTH 76.3 pg/ml at diagnosis
Bhadada et al. [18]	2008	14	Single parathyroid adenoma in 85.7%, 1 patient with four-gland hyperplasia and 1 MEN-1. Main reported symptoms were bone disease, recurrent nephrolithiasis, and pancreatitis. Mean serum Ca^++^: 2.77 mmol/l; mean P 0.9 mmol/l; mean serum PTH 781 pg/ml at diagnosis
Libansky et al. [19]	2008	10	Pediatric cohort with parathyroid adenoma and 1 ectopic adenoma. Mean serum Ca^++^: 3.21 mmol/l; mean serum PTH 217.6 pg/ml at diagnosis
Mallet E [4]	2008	55	31 adenomas and 11 hyperplasias underwent surgery. Medical management (i.v. diphosphonates) in 11 neonates. Mean serum Ca^++^: 3.64 mmol/l; mean serum P 1.3 mmol/l; mean serum PTH 536 pg/ml at diagnosis
Al-shanafey et al. [20]	2010	5	Surgical management in neonates with severe hyperparathyroidism, all symptomatic with lethargy, poor feeding, and irritability. In all patients, surgical treatment was curative. Mean serum Ca^++^: 3.84 mmol/l; mean serum PTH 3607 pg/ml at diagnosis
George et al. [5]	2010	15	Single parathyroid adenoma in 100% of patients. Main reported symptoms were bone pain, fractures, proximal myopathy, and renal calculi; 33.3% had postoperative HBS. Mean serum Ca^++^: 3.35 mmol/l; mean serum P 0.98 mmol/l; mean serum PTH 801 pg/ml at diagnosis; mean adenoma weight 3.84 gr
Shah et al. [21]	2012	19	Pediatric cohort with adenoma, presenting mostly bone pain (68%), weakness (68%), or fractures (52.6%)
Li et al. [22]	2012	12	Parathyroid adenoma in 100% (4/12 ectopic adenoma), presenting mostly urinary and bone tissue impairment. Mean serum Ca^++^: 3.82 mmol/l; mean serum P 1.18 mmol/l; mean serum PTH 1016 pg/ml at diagnosis
Belcher et al. [8]	2013	230	Literature review of studies regarding PHPT in the youth and adolescents. Single adenomas in 80% of patients, multiple-gland hyperplasia in 16.5% (MGH), double adenomas in 0.9%, and normal parathyroid gland in 2.6%. Of MGH, 50% were MEN I, MEN II, or familial non-MEN. Tc(99m)-sestamibi and ultrasound were 86% (37/43) and 74.5% (70/94) sensitive
Burke et al. [27	2013	19	Study aiming to enhance the radioguided parathyroidectomy. Adenoma in 74% and hyperplasia in 26%. No complications were noted in the pediatric patients after surgery. Mean serum Ca^++^: 3.05 mmol/; mean serum PTH 177 pg/ml at diagnosis. Mean serum Ca^++^: 2.35 mmol/; mean serum PTH 33 pg/ml after the surgery. Mean adenoma weight 0.44 gr
Alagaratnam S and Kurzawinski [9]	2014	29	Pediatric cohort with adenoma presenting mostly gastrointestinal symptoms (41%) and skeletal manifestations (20.7%)
Roizen and Levine [6]	2014	268	A meta-analysis comparing biochemical profiles in the youth and adults: greater hypercalcemia and hypercalciuria in youths at similar concentrations of serum intact PTH. Mean serum Ca^++^: 3.2 mmol/; mean serum P 0.9 mmol/l; mean serum PTH 331 pg/ml; mean ALP 995 UI/l at diagnosis. Mean gland weight 2 gr
Mancilla et al. [23]	2017	16	Pediatric cohort with parathyroid and thymic (2/16) adenoma, mostly symptomatic (75%). Mean serum Ca^++^: 3.02 mmol/; mean serum PTH 177.3 pg/ml at diagnosis
Lou et al. [11]	2017	40	Pediatric cohort with different patterns at diagnosis; increasing rate of diagnosis in asymptomatic subjects, higher postoperative complications, and disease recurrence in patients with positive familial history. Mean serum Ca^++^: 2.91 mmol/; mean serum PTH 152.5 pg/ml at diagnosis
Vannucci et al. [24]	2018	22	Pediatric cohort with MEN-1 mutation presenting PHPT in 50% of cases, mostly asymptomatic (10/11)
Saponaro et al. [25]	2018	31	Young and adult population with PHPT: significantly lower PTH, higher serum in the younger group. Nephrolithiasis, fragility, fracture, and densitometric parameter rates did not differ between groups. Mean serum Ca^++^: 2.73 mmol/; mean serum P 0.77 mmol/l; mean serum 25-hydroxyvitamin D 19.6 ng/ml, mean serum PTH 111 pg/ml; mean ALP 232 UI/l at diagnosis
Wang et al. [7]	2018	59	Pediatric cohort reporting bone pain as the most common manifestation, high rate of rickets (45.8%) compared to adults (23.7%), and an important correlation to short stature. Hypercalciuria, more frequent in pediatrics, hypophosphatemia, and urolithiasis among adults. Mean serum Ca^++^: 3.01 mmol/; mean serum PTH 177 pg/ml; mean serum ALP 374 UI/l; mean serum 25-hydroxyvitamin D 12.9 ng/ml at diagnosis
Rampp et al. [26]	2020	86	Pediatric cohort with parathyroid and thymic (22/86) adenoma presenting systemic and neurocognitive symptoms in 64% and nephrolithiasis in 20%. Mean serum Ca^++^: 2.93 mmol/; mean serum PTH 110 pg/ml at diagnosis. Mean gland weight 0.3 gr. Mean serum Ca^++^: 2.42 mmol/l after the surgery
Jovanovic et al. [3]	2020	14	Adults and youth comparison; bone disease in the youth (42.9%) and asymptomatic disease in adults (39.3%). Preoperative serum calcium and PTH significantly higher in the youth than in adults. Mean serum Ca^++^: 3.47 mmol/; mean serum P 0.8 mmol/l; mean serum PTH 572.6 pg/ml at diagnosis. Mean serum Ca^++^: 2.42 mmol/; mean serum P 0.92 mmol/l; mean serum PTH 22.8 pg/ml; 25-hydroxyvitamin D 39.5 ng/ml after the surgery

## Data Availability

The presented data are available on request from the corresponding author.
